# Differential Responses of Invasive Trees *Ailanthus altissima* Mill. Swingle and *Fraxinus americana* L. to Soil Phosphorus Availability

**DOI:** 10.3390/plants14203204

**Published:** 2025-10-18

**Authors:** Marijana Milutinović, Danijela Đunisijević-Bojović, Vuk Maksimović, Ljiljana Kostić Kravljanac, Jasmina Popović, Marija Marković

**Affiliations:** 1 Faculty of Forestry, University of Belgrade, Kneza Višeslava 1, 11030 Belgrade, Serbia; marijana.milutinovic@sfb.bg.ac.rs (M.M.) marija.markovic@sfb.bg.ac.rs (M.M.); 2 Institute for Multidisciplinary Research, University of Belgrade, Kneza Višeslava 1, 11030 Belgrade, Serbialjkostic@imsi.bg.ac.rs (L.K.K.)

**Keywords:** phosphorus availability, rhizoboxes, rhizosphere, biomass production, root exudates, lignin content, malate, nutrient acquisition, invasive species

## Abstract

The availability of phosphorus in the soil has a key role in plant physiological processes, particularly in the adaptive responses of invasive species. This study examined how contrasting soil phosphorus concentrations (low: 9 mg/kg and adequate: 27 mg/kg) influence biomass production, lignin and extractive content, P concentration in leaves, and root exudate composition in two invasive species, *Ailanthus altissima* and *Fraxinus americana*. Seedlings were grown in rhizoboxes filled with alkaline soils of two types. Adequate phosphorus concentration increased both aboveground and root biomass in the examined species, while low phosphorus significantly reduced biomass, especially in the aboveground parts, which were 3 to 4 times smaller compared to plants grown under adequate conditions. Low phosphorus concentration increased lignin and extractive content in the stem. Root exudate analysis revealed that low phosphorus availability enhanced the secretion of malate in both species. *Ailanthus altissima* exhibited higher malate concentrations in root exudates compared to *Fraxinus americana* under both phosphorus conditions. *Ailanthus altissima* is more competitive than *Fraxinus americana* on low-phosphorus alkaline soils. The results reveal how *Ailanthus altissima* and *Fraxinus americana* adapt to varying soil phosphorus levels, aiding the development of strategies to manage these invasive species and preserve ecosystem stability.

## 1. Introduction

Phosphorus availability in soil plays a fundamental role in plant physiological responses, nutrient uptake strategies, and overall adaptability. Alongside soil pH, it directly influences the bioavailability of essential nutrients and affects key metabolic and physiological processes. The ability of plant species to adapt to variable and often subadequate soil conditions is central to their ecological success, including invasiveness.

Invasive plant species are recognized as one of the leading drivers of biodiversity loss worldwide. They can significantly alter native plant communities, semi-natural ecosystems, or man-made ones (agroecosystems, etc.), disrupt ecological interactions, and change key soil properties, including nutrient availability, water retention, and microbial dynamics [1,2,3]. Invasive species, by definition, are non-native organisms that establish and spread in natural or semi-natural ecosystems, often outcompeting native biodiversity due to high phenotypic plasticity and adaptability [4,5]. According to IUCN, data based on DAISIE, 10,961 taxa of foreign invasive species have been recorded in Europe, and among the most aggressive are *Ailanthus altissima*, *Ambrosia artemisiifolia*, and *Robinia pseudoacacia* [6,7]. In Serbia, *Ailanthus altissima* (*Simaroubaceae*), native to Southeast Asia, is listed as a highly invasive species, while *Fraxinus americana* (*Oleaceae*), native to Southeast Asia, is considered sporadically invasive [8]. In native China, *Ailanthus altissima* is often found in calcareous areas [9], and it is highly tolerant of nutritionally poor, calcareous, and very alkaline soils [10], while *Fraxinus americana* can tolerate alkaline soils, but grows best in slightly acidic, neutral, or slightly alkaline soil pH [11].

The success of invasive species is often linked to their ability to exploit environmental resources more efficiently than native species, especially under subadequate conditions [12]. One of the key resources that often limits plant productivity is phosphorus (P), an essential macronutrient involved in fundamental biological processes such as energy transfer, signal transduction, and macromolecule biosynthesis [13,14,15,16]. Invasive species can successfully colonize and thrive in alkaline soils by adapting to the low availability of certain nutrients caused by the high pH of alkaline soil. In alkaline soils, phosphorus is poorly available, which further affects the deficiency of other nutrients such as Fe, Cu, Zn, Ca, and Mn [17,18,19]. As a result, plants have evolved a suite of adaptive mechanisms to cope with P-deficiency, including changes in root, increased expression of phosphate transporters, enhanced lignification, and the release of carboxylates and other root exudates that mobilize bound phosphorus in the rhizosphere [16,20,21].

Root exudates composed of amino acids, sugars, organic anions, phenolics, and other secondary metabolites play a central role in plant–soil–microbe interactions [22]. Under phosphorus deficiency, many plant species increase the secretion of low molecular weight organic anions such as malate, oxalate, and citrate, which facilitate phosphorus solubilization from insoluble forms [16,23]. These compounds can also have allelopathic effects, altering microbial communities or inhibiting the germination and establishment of competing plant species [24,25,26,27]. Invasive species may exploit such traits to enhance their competitiveness, particularly in nutrient-poor environments [2,13,28].

Lignin is one of the main components of the plant cell wall, a natural phenolic polymer with a complex composition and structure. As such, it increases the rigidity and plasticity of plant cell walls, its hydrophobic properties, and stimulates the transport of minerals through the vascular bundles of plants [29,30]. The interplay between lignin accumulation and nutrient availability, particularly phosphorus, remains poorly understood in invasive species [31].

The aim of this study is to investigate how contrasting phosphorus concentrations in alkaline soils affect biomass production, lignin, and extractive content in aboveground tissues, concentration of P in leaves, and the composition of root exudates in two invasive tree species, *Ailanthus altissima* and *Fraxinus americana*. By exploring these physiological responses, this study provides insights into the adaptive strategies that enable invasive species to thrive under nutrient-limited conditions. These findings may contribute to the development of more effective invasive species management and soil restoration strategies.

## 2. Results

### 2.1. Soil Chemical Properties and Phosphorus Content in the Soil

The phosphorus content in the soil sampled from Fruška gora (9 mg/kg—designated as Soil 1) was low (Table 1), whereas the soil sampled from the Arboretum of the Faculty of Forestry, University of Belgrade (27 mg/kg, designated as Soil 2) demonstrated adequate phosphorus content (Table 1).

Chemical analysis of the sampled soil (Soil 1 and Soil 2) revealed that they exhibit alkaline reactions with distinct available phosphorus concentrations (low in Soil 1 and high in Soil 2 according to Oisen phosphorus classification).

### 2.2. Biomass Production in Response to Soil Phosphorus Availability

**Figure 1:**
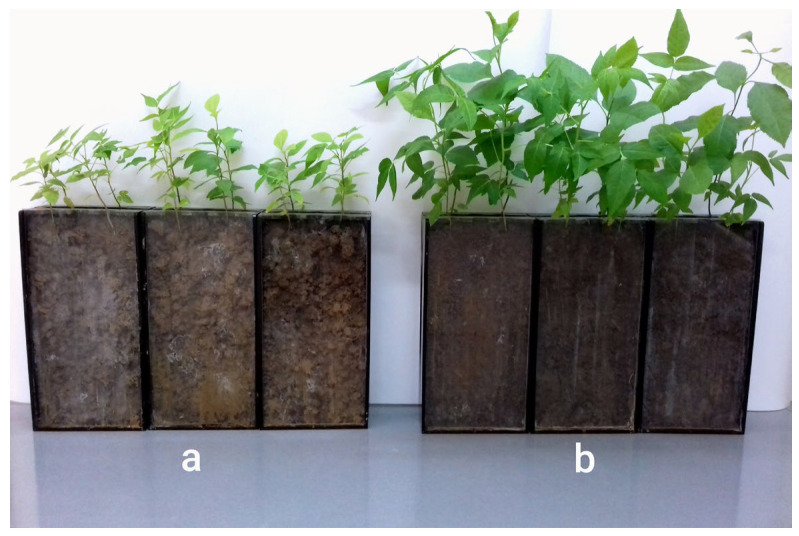
Biomass production in *Ailanthus altissima* and *Fraxinus americana* under different soil phosphorus conditions: (**a**) Plants grown in soil with low phosphorus concentration (Soil 1; 9 mg/kg) and (**b**) Plants grown in soil with adequate phosphorus concentration (Soil 2; 27 mg/kg).

The results indicate that the phosphorus content in the soil significantly influenced the biomass production of the aboveground parts of *Ailanthus altissima* and *Fraxinus americana* in the experimental rhizoboxes, with a lesser effect observed on root biomass (Figure 1).

The average mass of the aboveground parts of *Ailanthus altissima* (0.63 g) and *Fraxinus americana* (0.70 g) in soil with adequate phosphorus content (Table 2) is 3–4 times higher than the aboveground parts of the same species, grown in soil with low phosphorus content (*Ailanthus altissima* 0.23 g and *Fraxinus americana* 0.18 g) (Table 2).

Phosphorus concentration did not influence root biomass of observed species (Table 2). Also, based on the obtained results, it is noticed that the mass of the aboveground part between *Ailanthus altissima* and *Fraxinus americana* grown in Soil 1 and Soil 2 is approximately the same, as well as the root mass of the tested plants grown in Soil 1 and Soil 2 (Table 2). The adequate phosphorus concentration resulted in a root biomass of *Fraxinus americana* that was approximately twice as high as in *Ailanthus altissima* (Table 2). The root/shoot ratio varied depending on species and phosphorus availability (Table 2). In *Fraxinus americana*, the root/shoot ratio was higher under low phosphorus supply (0.72), while it decreased under adequate phosphorus conditions (0.27). Similarly, *Ailanthus altissima* exhibited a higher root/shoot ratio under low phosphorus (0.44), which declined to 0.14 under adequate phosphorus availability. Phosphorus deficiency significantly increased the root/shoot ratio in both species, reflecting a shift in biomass allocation characterized by a relatively greater investment in root biomass compared to shoot biomass under low phosphorus conditions.

### 2.3. Lignin and Extractive Content in Plant Tissues

**Figure 2:**
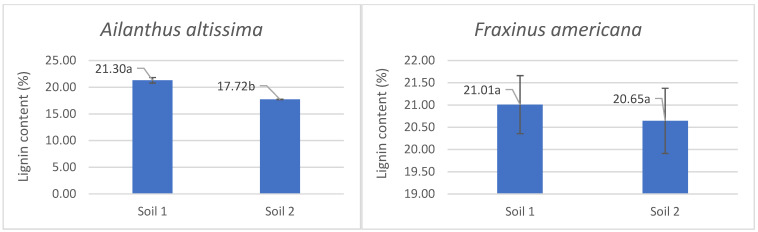
The average percentage content of lignin with standard errors and indication of statistically significant difference (a, b) according to ANOVA and Fisher’s least significant difference (LSD) test at the 95% confidence level (*p* < 0.05) in the aboveground part of the *Ailanthus altissima* and *Fraxinus americana* grown in soil with low (9 mg/kg—Soil 1) and adequate (27 mg/kg—Soil 2) degrees of phosphorus concentration.

Analysis of lignin content in the aboveground parts of plants grown under different phosphorus availability conditions showed that both *Ailanthus altissima* and *Fraxinus americana* showed higher lignin content when grown in soil with low phosphorus availability (Soil 1), compared to soil with adequate phosphorus levels (Soil 2) (Figure 2). Specifically, the lignin content of *Ailanthus altissima* was 21.30% in Soil 1 and significantly decreased to 17.72% in Soil 2, which was statistically significant (*p* < 0.05). In *Fraxinus americana*, lignin values were 21.01% in Soil 1 and 20.65% in Soil 2, with no statistically significant difference observed. These findings indicate that *Ailanthus altissima* responds to low phosphorus availability with increased lignification, while *Fraxinus americana* maintains relatively stable lignin content regardless of soil phosphorus conditions.

**Figure 3:**
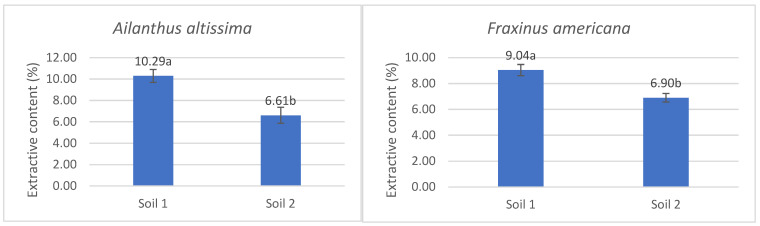
The percentage content of extractives with standard errors in the aboveground part of the species *Ailanthus altissima* and *Fraxinus americana* grown in soil with low (Soil 1) and adequate (Soil 2) degrees of phosphorus concentration in the soil. The statistically significant difference, indicated by letters a and b, was determined using ANOVA and Fisher’s least significant difference (LSD) test at the 95% confidence level (*p* < 0.05).

By analyzing the extractive content in the aboveground parts of the plants, it was determined that its values were significantly higher in *Ailanthus altissima* and *Fraxinus americana* under conditions of low phosphorus availability in the soil (Soil 1), compared to adequate phosphorus availability (Soil 2) (Figure 3). In *Ailanthus altissima*, the mean value in Soil 1 was 10.29%, whereas in Soil 2 it was 6.61%, indicating a statistically significant difference (*p* < 0.05). Similarly, *Fraxinus americana* showed an average value of 9.04% in Soil 1 and 6.90% in Soil 2, also confirming a significant difference depending on the phosphorus treatment (Figure 3). These results indicate a significant impact of phosphorus availability on extractive biosynthesis and accumulation in both species, with both exhibiting reduced values under adequate phosphorus conditions.

### 2.4. Root Exudation of Organic Anions Under Contrasting Phosphorus Availability in Soil

**Figure 4:**
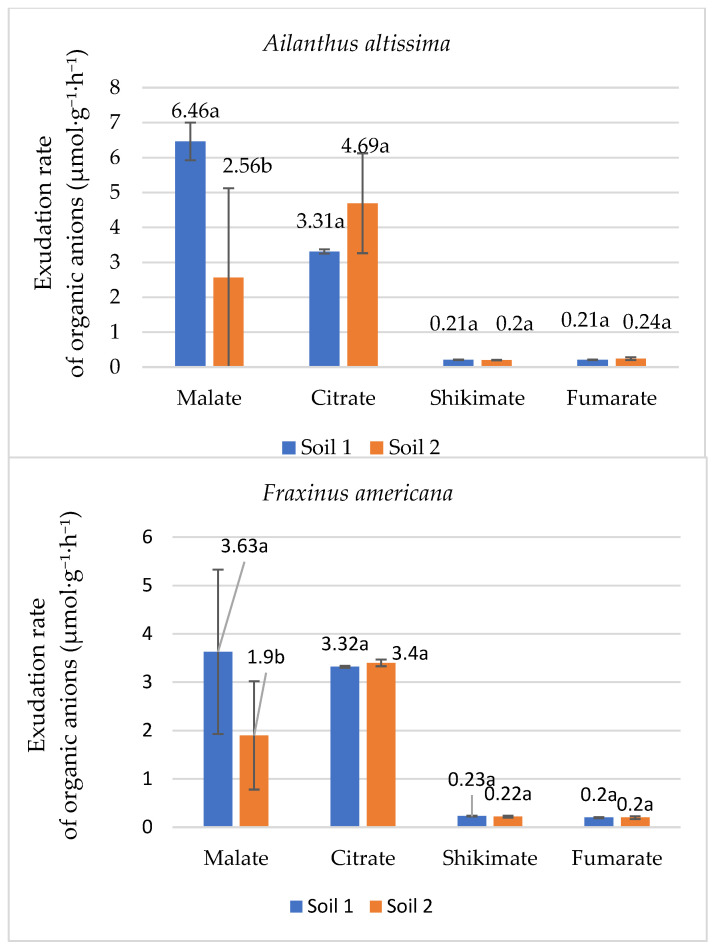
Mean exudation rate (µmol·g^−1^·h^−1^ ± SE) of organic anions in root exudates of the species *Ailanthus altissima* and *Fraxinus americana* grown in soil with low (Soil 1) and adequate (Soil 2) degrees of phosphorus concentration in the soil. Different letters indicate statistically significant differences (*p* < 0.05) based on ANOVA and the LSD test.

The exudation of organic anions such as malate, citrate, shikimate, and fumarate plays a critical role in plant adaptation to phosphorus (P) deficiency, facilitating nutrient uptake from the soil. Therefore, the research included the examination of these organic anions. Malate, in particular, showed the most pronounced response to phosphorus availability, highlighting its importance in nutrient acquisition mechanisms (Figure 4).

As indicated in Figure 4, phosphorus availability in the soil significantly influences the concentration of these organic anions in the root exudates of both *Ailanthus altissima* and *Fraxinus americana*. Specifically, *Ailanthus altissima* showed a substantial increase in malate exudation rate in response to low phosphorus conditions, with values reaching 6.46 µmol g^−1^ h^−1^ in soil with low phosphorus (Soil 1), compared to 2.56 µmol g^−1^ h^−1^ in adequate phosphorus conditions (Soil 2). This difference was statistically significant, indicating that low phosphorus availability strongly stimulates malate exudation in this species. This represents an approximate threefold increase in malate exudation under phosphorus-deficient conditions. Similarly, in *Fraxinus americana*, the malate exudation rate in root exudates was higher under low phosphorus conditions (3.63 µmol g^−1^ h^−1^) than in adequate phosphorus conditions (1.90 µmol g^−1^ h^−1^), demonstrating a doubling of malate release under phosphorus-deficient conditions. In addition to malate, citrate, shikimate, and fumarate were also measured, but their concentrations exhibited smaller variations between the two phosphorus concentrations in soil in both species. For example, citrate concentrations were slightly higher in *Ailanthus altissima* grown in adequate phosphorus soil (4.69 µmol g^−1^ h^−1^) compared to low phosphorus soil (3.31 µmol g^−1^ h^−1^). In comparison, *Fraxinus americana* showed only minor differences in citrate concentrations between the two soil types (3.32 µmol g^−1^ h^−1^ under low phosphorus and 3.40 µmol g^−1^ h^−1^ under adequate phosphorus). Shikimate and fumarate showed similar values, with minor changes that did not reflect significant changes in response to phosphorus availability. These results indicate that malate is the most responsive organic anion to changes in phosphorus availability, playing a key role in the adaptive strategies of both plant species under phosphorus-deficient conditions.

### 2.5. Leaf Phosphorus Content Under Contrasting Soil P Availability

**Figure 5:**
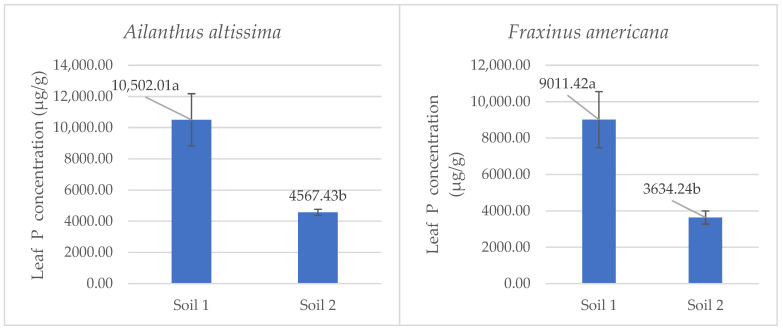
Mean phosphorus concentrations (µg·g^−1^ ± SE) in leaf tissue of the species *Ailanthus altissima* and *Fraxinus americana* grown in soil with low (Soil 1) and adequate (Soil 2) phosphorus availability. Different letters indicate statistically significant differences (*p* < 0.05) based on ANOVA and the LSD test.

The analysis of phosphorus concentration in leaves revealed significant differences between low and adequate soil phosphorus availability for both *Ailanthus altissima* and *Fraxinus americana* (Figure 5).

In *Ailanthus altissima*, plants grown in low-phosphorus availability (Soil 1) had a statistically significant (*p* < 0.05) higher phosphorus concentration in leaves (10,502.01 µg/g) compared to those grown in adequately supplied soil (Soil 2), which showed an average concentration of 4567.43 µg/g. Similarly, *Fraxinus americana* plants grown under phosphorus-deficient conditions (Soil 1) had a significantly higher phosphorus concentration in leaves (9011.42 µg/g) than those grown in phosphorus-adequately supplied soil (Soil 2), which contained 3634.24 µg/g. These results highlight the pronounced physiological adaptations of both invasive species to phosphorus deficiency in alkaline soils. Notably, *Ailanthus altissima* demonstrated a stronger adaptive response, maintaining a higher accumulation of phosphorus in leaf tissues under low P conditions. This adaptive mechanism aligns with the observed increase in malate exudation rate from roots, emphasizing the role of malate in phosphorus mobilization and in enhancing plant tolerance to nutrient stress.

## 3. Discussion

### 3.1. The Role of Phosphorus Availability on Biomass Production

The soil pH, calcium carbonate level, mineral composition, and organic matter are factors that determine available phosphorus content in the soil. The soil pH, from slightly alkaline (Soil 2) to alkaline (Soil 1), strongly influences phosphorus availability. In soils with a pH higher than 8 (Soil 1), available phosphorus is decreased. This effect is due to the increased reduction of Pi with calcium. The lowest solubility of Pi is in calcareous soils with a pH higher than 8. Considering this fact, we have analyzed the influence of contrast phosphorus availability as the main factor affecting plant responses.

The data clearly show that phosphorus availability is a key factor in biomass production, with higher phosphorus concentrations promoting greater growth. This effect was especially pronounced in *Ailanthus altissima*, which exhibited strong resilience to low phosphorus supply. The implications of these results may extend beyond the specific species studied. Understanding the role of phosphorus in plant growth can aid in developing more efficient soil management strategies, particularly in phosphorus-deficient soils. It can be concluded that *Ailanthus altissima* demonstrates remarkable resistance to low phosphorus concentration in the soil, as shown in Table 2. Plass [32] also reports that *Ailanthus altissima* is a highly resistant species, showing significant biomass production even under low phosphorus conditions. This further supports our hypothesis that phosphorus availability influences biomass production. Phosphorus deficiency increased the root/shoot ratio in both species, indicating a shift in biomass allocation with relatively greater investment in root growth, which may enhance nutrient uptake efficiency under low phosphorus conditions.

### 3.2. Changes in Lignin and Extractive Content Depending on Phosphorus Availability

As one of the key components of plant cell walls, lignin provides mechanical support and protection against environmental stressors [33,34]. The increased lignin content in plants grown under low phosphorus conditions is probably a consequence of a physiological response aimed at improving structural integrity and resistance to environmental stress. Future research should investigate how phosphorus availability affects plant growth and nutrient cycling in natural ecosystems. Additionally, examining the impact of varying phosphorus levels on other tree species may provide deeper insights into different plant nutrient strategies. Similarly, the observed increase in extractive content under low phosphorus conditions reflects a well-documented plant adaptive mechanism whereby secondary metabolite synthesis is enhanced in response to nutrient stress [35,36]. Extractive compounds, which include phenolics, lipids, and other low-molecular-weight compounds, play crucial roles in protecting plants against oxidative damage, herbivory, and pathogen attack, as well as in modulating cellular signaling pathways [37]. The significant accumulation of extractives in both *Ailanthus altissima* and *Fraxinus americana* under phosphorus deficiency suggests an upregulated defense metabolic response aimed at reinforcing nutritive stress tolerance. This is consistent with the existing concept that environmental factors stimulate secondary metabolite production as a survival strategy [38]. Also, with increased phosphorus content, the lignin content decreases [39]. The increase in lignin and extractive contents indicates that these species employ both structural (lignin) and chemical (extractives) adaptations to cope with low phosphorus availability. Such multifaceted responses highlight the importance of phosphorus as a key regulator of plant secondary metabolism. Further studies focusing on the biochemical pathways and ecological implications of these changes would enhance understanding of plant resilience under nutrient limitation.

### 3.3. Organic Anion Root Exudation as a Response to Phosphorus Deficiency

As a response to environmental stress, such as nutrient deficiency, plants release root exudates to adapt and enhance nutrient acquisition and improve survival [40]. In this study, the concentrations of key organic anions were measured to assess species-specific responses to phosphorus availability. A notable difference was observed in the exudation rate of malate between *Ailanthus altissima* and *Fraxinus americana* grown in phosphorus-deficient soil compared to soil with adequate phosphorus availability. For instance, *Ailanthus altissima* significantly increased malate exudation under low phosphorus conditions (6.46 µmol g^−1^ h^−1^) compared to adequate phosphorus (2.56 µmol g^−1^ h^−1^), indicating a strong adaptive response. Additional mechanisms, such as increased phosphatase activity, have also been reported in response to phosphorus stress [40,41]. These findings suggest that root exudation patterns vary depending on the species and environmental conditions. Root exudate composition and rate are known to be species-specific, with woody plants generally releasing higher amounts of exudates than herbaceous species [41]. The results further indicate that phosphorus availability significantly influences the concentrations of organic anions in root exudates of *Ailanthus altissima* and *Fraxinus americana*. *Ailanthus altissima* showed a stronger adaptive response to phosphorus deficiency, with malate exudation rate tripling under low phosphorus conditions (from 2.56 µmol g^−1^ h^−1^ in Soil 2 to 6.46 µmol g^−1^ h^−1^ in Soil 1). In contrast, *Fraxinus americana* exhibited a less pronounced increase, with malate exudation rate doubling under low phosphorus conditions (from 1.90 µmol g^−1^ h^−1^ in Soil 2 to 3.63 µmol g^−1^ h^−1^ in Soil 1). The citrate exudation rate in *Fraxinus americana* showed minimal variation between contrast phosphorus availability (3.32 µmol g^−1^ h^−1^ in Soil 1 and 3.40 µmol g^−1^ h^−1^ in Soil 2). These results highlight the stronger adaptive capacity of *Ailanthus altissima* to phosphorus deficiency through enhanced exudation of malate, compared to *Fraxinus americana*, underscoring the species-specific nature of root exudation in response to nutrient availability. Additionally, the allelopathic effects of these exudates may contribute to the invasive potential of these species by influencing microbial communities or suppressing native competitors [42].

### 3.4. Leaf Phosphorus Accumulation in Response to Contrasting Soil P Availability

Phosphorus (P) is an essential macronutrient involved in numerous physiological and metabolic processes, including energy transfer via ATP, nucleic acid synthesis, and membrane integrity [14,16,18]. In phosphorus-deficient soils, plants often develop adaptive strategies to enhance P acquisition, including morphological and biochemical changes. One reliable indicator of P uptake efficiency is the concentration of phosphorus in leaf tissue, as it reflects both the plant’s access to soil P and its internal allocation mechanisms [18,43]. In this study, both *Ailanthus altissima* and *Fraxinus americana* exhibited significantly higher concentrations of phosphorus in their leaf tissues when grown in phosphorus-deficient alkaline soils (Soil 1) compared to plants grown in soils with an adequate phosphorus supply (Soil 2). Specifically, *Ailanthus altissima* accumulated 10,502.01 µg/g of P in leaves under low P conditions, more than double the concentration observed under adequate P concentration (4567.43 µg/g). Similarly, *Fraxinus americana* showed a notable increase in leaf P from 3634.24 µg/g to 9011.42 µg/g under the same contrasting soil conditions, with statistically significant differences. These results point to improved phosphorus mobilization and absorption strategies. One such mechanism is increased excretion of organic anions such as malate, which facilitates the mobilization of otherwise unavailable phosphorus bound to calcium or other cations in alkaline soils [19,44]. Our findings show a threefold increase in malate excretion in *Ailanthus altissima* and a twofold increase in *Fraxinus americana* under phosphorus-deficient conditions, supporting this explanation. Invasive species such as *Ailanthus altissima* are often characterized by high nutrient use efficiency and phenotypic adaptability, enabling them to thrive in resource-poor environments [16,45]. The increased malate excretion observed under phosphorus-deficient conditions likely increased phosphorus mobilization in the rhizosphere, contributing to higher leaf phosphorus concentrations and supporting higher biomass production in both species, especially in *Ailanthus altissima*, which makes it the dominant species.

## 4. Materials and Methods

### 4.1. Plant Material and Growing Conditions

A set of studies was conducted to examine the ecophysiological adaptive strategies of the invasive plant species *Ailanthus altissima* (Mill.) Swingle and *Fraxinus americana* L. to phosphorus deficiency in alkaline soil. For this purpose, both plant species were grown in rhizoboxes filled with soil characterized by contrasting levels of phosphorus availability. The seedlings of *Ailanthus altissima* and *Fraxinus americana* were cultivated in soil culture within 12 rhizoboxes for a period of 3 months.

The seeds from both species are taken from the same respective trees: Fraxinus americana from the locality in Belgrade and Ailanthus altissima from the Erdevik locality. The experiment was conducted in the case of three-month-old seedlings, in a separate chamber of the greenhouse (Faculty of Forestry, Belgrade University). The rhizoboxes, dimensions of 30 × 15 × 4.5 cm, made of plexiglass with a transparent front panel that serves as a cover, enable the examination of plant rhizosphere processes. The rhizoboxes were filled with two alkaline soils collected from distinct locations in Serbia: Fruška gora, Erdevik (Soil 1), and the Arboretum of the Faculty of Forestry, Belgrade (Soil 2). These soils differed in their concentrations of available P.

### 4.2. Soil Sampling and Determination of Chemical Properties

Soil samples were collected from the aforementioned locations (Fruška gora, Erdevik, and the Arboretum of the University of Belgrade, Faculty of Forestry) using standard sampling procedures. During sampling, both soils were characterized as loamy-clayey using the field method. The samples were dried and sieved through a 2 mm opening sieve to remove larger particles and organic debris. These prepared soil samples were subsequently used for the experiment and laboratory analyses to determine their chemical properties and nutrient content.

The chemical properties of the soils used in the experiment were analyzed to determine their nutrient status and key characteristics in Table 1. The analysis included pH, electrical conductivity (EC), cation exchange capacity (CEC), organic matter content, calcium carbonate content, and concentrations of essential macronutrients and micronutrients.

### 4.3. Experimental Design

**Figure 6:**
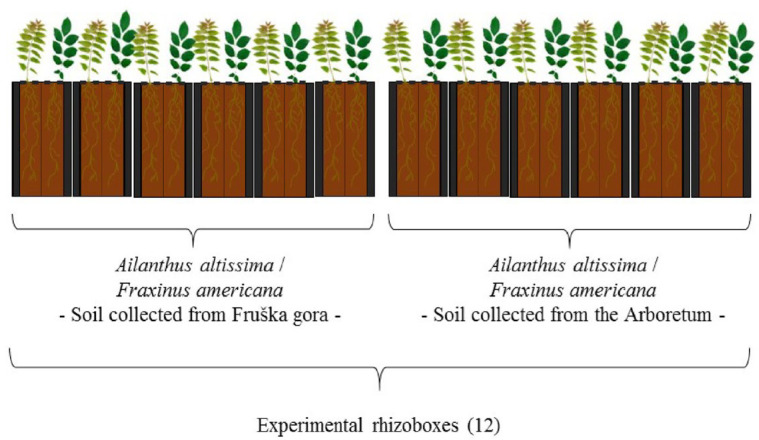
Experimental setup showing rhizoboxes used to cultivate *Ailanthus altissima* and *Fraxinus americana* under two phosphorus availability conditions: (**left**) Rhizoboxes filled with soil of low phosphorus availability (Soil 1, Fruška gora); (**right**) Rhizoboxes filled with soil of adequate phosphorus availability (Soil 2, Arboretum).

A total of twelve rhizoboxes were used in the experimental setup. Each rhizobox contained one seedling of *Ailanthus altissima* and one seedling of *Fraxinus americana*, allowing both species to grow simultaneously under identical soil and other conditions. Six rhizoboxes were filled with soil collected from Fruška gora, characterized by low phosphorus availability, while the remaining six were filled with soil from the Arboretum, which exhibited adequate phosphorus levels (Figure 6). Such an experimental design enabled the assessment of species-specific physiological responses, root exudation, lignin and extractive content, production of biomass, P content in leaves, and rhizosphere interactions in relation to soil phosphorus status.

### 4.4. Laboratory and Experimental Analyses

Laboratory and experimental analyses determined the chemical characteristics of the soil, biomass production, composition and content of carboxylates in root exudates, phosphorus concentration in leaves, lignin, and extractive content in the aboveground part (stem) of the invasive species *Ailanthus altissima* and *Fraxinus americana*.

#### 4.4.1. Soil Analysis

The soil pH and EC were measured in both H_2_O (soil: solution ratio of 1:2.5). Calcium carbonate (CaCO_3_) content was determined with the Scheibler volumetric method. The concentrations of total C, N, and S were determined by dry combustion methods using a CHNS Elemental Analyzer Vario microcube (Elementar Analysensysteme GmbH, Hanau, Germany). Available P was extracted by the Olsen method (0.5 M NaHCO_3_, pH 8.5), and the P concentration was measured by the colorimetric molybdenum blue method at 882 nm.

Plant available microelements (Cu, Fe, Zn, Mn, Ni, Mo) were extracted by the DTPA-TEA solution (buffered at pH 7.3; soil: solution ratio of 1:5). Plant available B was extracted using the Sorbitol method. Plant available K, Ca, and Mg were extracted and determined using the ammonium acetate extraction method (shaking 5 min, soil: solution ratio of 1:5). CEC was calculated. Concentration of available elements (except for P) was determined by inductively coupled plasma optical emission spectrometry (ICP-OES; Spectro-Genesis EOP II, Spectro Analytical Instruments GmbH, Kleve, Germany).

#### 4.4.2. Biomass Production

The dry biomass of the aboveground parts and roots of *Ailanthus altissima* and *Fraxinus americana* was measured after cultivation in rhizoboxes. The plant samples were dried at a temperature between 60 °C and 70 °C until they reached a constant weight, which was verified by two consecutive measurements with identical values. The dry mass was measured using a high-precision analytical balance to ensure the accuracy of the results.

#### 4.4.3. Lignin and Extractive Content in the Aboveground Plant Parts

Plant tissue samples were prepared by grinding with a hammer mill (Culatti AG micro mill DFH 48, Schweiz, Switzerland) to a particle size of 0.5–1 mm [46]. The moisture content of the samples was determined by drying to a constant mass at 105 ± 2 °C [47]. The samples were prepared for the determination of lignin and extractive content by extraction with neutral organic solvents to remove non-structural compounds such as lipids, pigments, and low-molecular-weight phenolics according to Standard Test Method [48]. A Soxhlet extractor was used for this continuous extraction process, using an ethanol-toluene mixture (2:1, *v*/*v*) as the solvent. After eight hours of extraction, the remaining extract-free biomass was subjected to a two-step acid hydrolysis process using concentrated (72% H_2_SO_4_) in the first step and dilute sulfuric acid (3% H_2_SO_4_) in the second step [49]. During this process, the structural carbohydrates (cellulose and hemicellulose) were hydrolyzed and dissolved, while lignin remained as an acid-insoluble residue. Additionally, the acid-soluble part of the lignin is determined by UV absorption (Evolution 300 UV-Vis spectrophotometer, Thermo Electron Corporation, Altrincham, UK) of the acid hydrolysate at 205 nm [50]. The lignin content was shown as the sum of acid-insoluble and acid-soluble lignin, and expressed as a percentage of the dry biomass. The extractive content was determined gravimetrically based on mass differences before and after extraction. The following parameters were measured: the mass of the empty extraction thimble, the mass of the thimble with the moist wood sample, the mass of the empty extraction cup, and the mass of the cup containing the collected extractives after solvent evaporation. Based on these measurements, the mass of the moist wood sample and the mass of the isolated extractives were calculated. After solvent evaporation, the mass of extractives was determined using an analytical balance. The extractive content was then calculated and expressed as a percentage of the dry wood mass.

#### 4.4.4. Collection of Root Exudates and Determination of Organic Acids

Root exudates were collected from intact root tips (0–20 mm) using sample application papers for electrophoresis (10 × 5 mm; SERVA Electrophoresis GmbH, Heidelberg, Germany) moistened with deionized water. The intact root tips were fixed between two small plastic sheets using clamps and covered with filter paper to prevent root exposure to light. After 1 h, the paper pieces with absorbed root exudates were extracted in a methanol: deionized H_2_O (1:3 *v*/*v*) mixture.

These samples were then analyzed using High-Performance Liquid Chromatography (HPLC) to separate and identify the organic acids. The separation was carried out on a Waters HPLC system with 1525 binary pumps, a thermostat, a 717+ autosampler, and a 2996 diode array detector set at 210 nm. Separation was performed on a 300 × 7.8 mm anion exchange column (Aminex HPX-87H, Bio-RadHercules, CA, USA) with 5 mmol L^−1^ H_2_SO_4_ as the mobile phase, using isocratic elution at 0.6 mL min^−1^ and 40 °C. Quantification was performed by the external standard method using pure standards of organic acids based on their concentration, retention time, and UV spectra. The data acquisition and spectral evaluation for peak confirmation were carried out by the Waters Empower 2 Software (Waters, Milford, MA, USA).

#### 4.4.5. Phosphorus Analysis in Leaf Tissue via Microwave Digestion and ICP-OES

After cultivation in rhizoboxes, the plant samples were dried at a temperature between 60 °C and 70 °C until they reached a constant mass, confirmed by two consecutive measurements with identical values. The dry mass of leaves was then determined using a high-precision analytical balance to ensure accuracy. Dried plant material was subsequently ground. Samples of approximately 0.2 g (or less) were subjected to microwave-assisted digestion using 3 mL of concentrated nitric acid (HNO_3_) and 2 mL of hydrogen peroxide (H_2_O_2_), performed in a Microwave Accelerated Reaction System (Model MARS 5, CEM Corporation, Matthews, NC, USA). This digestion method was used for the quantification of phosphorus (P). After that, concentrations in the digested solutions were determined using Inductively Coupled Plasma Optical Emission Spectrometry (ICP-OES), Spectro Genesis EOP II (Spectro Analytical Instruments GmbH, Kleve, Germany).

### 4.5. Statistical Analysis

Statistical analysis of the data was performed using the software packages STATGRAPHICS Plus (version 2.1) and Microsoft Excel 2013. Various statistical tests were applied to analyze the data, including the Analysis of Variance (ANOVA), Table of Means, Multiple Range Test (LSD), and Kruskal–Wallis test, depending on the type and distribution of the data. Significance was determined at a threshold of *p* < 0.05. Results are presented as mean ± standard error (SE), with homogeneous groups identified using the Multiple Range Test (LSD). Homogeneous groups were determined using one-way analysis of variance (ANOVA) and Multiple Range Test (Fisher’s least significant difference (LSD) test at the 95% confidence level (*p* < 0.05). Different letters (a, b, ab, c) indicate a significant difference. The letter “a” represents the statistically highest value, while the following letter “b” represents statistically lower values, and the letter “c” represents the lowest value.

## 5. Conclusions

This study clearly demonstrated that contrasting phosphorus concentrations significantly influenced key plant traits in two invasive woody species, *Ailanthus altissima* and *Fraxinus americana*, such as biomass production, lignin and extractive content, composition of root exudates, and P concentration in leaves.

Under low phosphorus conditions, both species exhibited reduced aboveground biomass. Aboveground biomass was 3–4 times greater in soils with adequate phosphorus content compared to phosphorus-deficient soils. Lignin content analysis revealed that soil phosphorus availability significantly affects lignin synthesis and accumulation in the aboveground parts of *Ailanthus altissima* and *Fraxinus americana*. Both species exhibited higher lignin content under low phosphorus conditions, suggesting that phosphorus deficiency triggers increased lignin production as an adaptive response to stress, potentially reinforcing cell walls. Moreover, the notable decrease in extractive content under adequate phosphorus suggests that phosphorus availability influences secondary metabolite accumulation, which may play a role in stress adaptation in these invasive species.

The study emphasized the importance of organic anion exudation in nutrient uptake under phosphorus-deficient conditions. Malate exudation was the most responsive, with exudation rates tripling in *Ailanthus altissima* and doubling in *Fraxinus americana* under low phosphorus concentration in soil. In contrast, citrate, shikimate, and fumarate exudation exhibited only minor variations between low and adequate phosphorus levels. According to the interpretation of available phosphorus concentration in alkaline soil, the concentration of 27 mgP/mg is considered optimal Olsen P [51]. These results highlight malate as the most responsive organic anion, a key indicator of plant adaptation to phosphorus stress, playing a central role in its mobilization and uptake. Our results showed that both *Ailanthus altissima* and *Fraxinus americana* accumulated significantly higher concentrations of phosphorus in their leaves when grown in low-phosphorus soil conditions, despite reduced phosphorus availability. These results were associated with increased malate concentration, which improved phosphorus mobilization and absorption. The ability to maintain elevated phosphorus levels in leaves under nutrient stress, particularly in *Ailanthus altissima*, also results in higher biomass production and reflects a strong adaptive response to phosphorus availability in alkaline soils.

The distinct adaptive strategies to low phosphorus availability in alkaline soils employed by the studied species may be triggered by the biological and ecological properties of the species, as they represent different plant families and have different geographic origins. *Ailanthus altissima* exhibited a more efficient adaptive response by integrating enhanced malate exudation, greater phosphorus accumulation in leaves, and relatively stable biomass production compared to *Fraxinus americana*. These traits indicate a higher phosphorus uptake efficiency and support its greater competitiveness and potential dominance in nutrient-limited, alkaline environments.

This study highlights the importance of investigating root-soil interactions, exudate composition, and nutrient distribution when assessing plant adaptability. The insights can lead to further research on nutrient use efficiency, sustainable soil management, and practical strategies for ecological control of invasive woody species.

## Figures and Tables

**Table 1:** Chemical characteristics of experimental soils.

Parameters	Soil 1	Soil 2
pH in H_2_O	8.2	7.4
EC in H_2_O (dS·m^−1^)	0.14	0.07
CEC (cmol_c_ kg^−1^)	27	22
Organic matter (%)	1.7	0.3
CaCO_3_ (%)	1.3	1
Total N (g kg^−1^)	1.0	0.5
Olsen P (mg kg^−1^)	9	27
Exchangeable K (mg kg^−1^)	167	189
Exchangeable Ca (mg kg^−1^)	4712	3414
Exchangeable Mg (mg kg^−1^)	360	512
Available B (mg kg^−1^)	0.84	0.35
Available Fe (mg kg^−1^)	16	35
Available Cu (mg kg^−1^)	1.4	1.55
Available Mn (mg kg^−1^)	16	9.6
Available Zn (mg kg^−1^)	1.56	0.15
Available Mo (mg kg^−1^)	0.034	0.030

**Table 2:** Mean dry mass (g ± SE) of shoot and root biomass and root/shoot ratio of *Ailanthus altissima* and *Fraxinus americana* grown in soils with adequate and low phosphorus availability. Different letters in exponent (a, b) of mean values indicate statistically significant differences (*p* < 0.05) based on ANOVA and LSD test.

Species Name	Type of Soil *	Part of the Plants (Mean Values **)		Root/Shoot Ratio
		Root	Shoot	
*Fraxinus americana*	Soil 2	0.19 ^a^ ± 0.06	0.70 ^a^ ± 0.16	0.27 ^b^ ± 0.11
	Soil 1	0.13 ^a^ ± 0.03	0.18 ^b^ ± 0.03	0.72 ^a^ ± 0.21
*Ailanthus altissima*	Soil 2	0.09 ^a^ ± 0.02	0.63 ^a^ ± 0.14	0.14 ^b^ ± 0.05
	Soil 1	0.10 ^a^ ± 0.03	0.23 ^b^ ± 0.04	0.44 ^a^ ± 0.15

* -Type of soil * Soil 1 represents low phosphorus concentration (9 mg/kg); Soil 2 represents adequate phosphorus concentration (27 mg/kg); -Mean values ** Mean values are presented as mean ± standard error. Homogeneous groups were determined using one-way analysis of variance (ANOVA) and Fisher’s least significant difference (LSD) test at the 95% confidence level (*p* < 0.05).

## Data Availability

The original contributions presented in this study are included in the article. Further inquiries can be directed to the corresponding author.
